# Alisol A 24‐acetate ameliorates osteoarthritis progression by inhibiting reactive oxygen species and inflammatory response through the AMPK/mTOR pathway

**DOI:** 10.1002/iid3.848

**Published:** 2023-05-08

**Authors:** Guosong Xu, Zhensen He, Yinping Liu

**Affiliations:** ^1^ Department of Orthopedics The First Hospital of Putian City Putian China

**Keywords:** alisol A 24‐acetate, inflammation, osteoarthritis, oxidative stress, reactive oxygen species

## Abstract

**Introduction:**

Osteoarthritis is a degenerative knee joint disease featured with articular cartilage degeneration and inflammation. Alisol A 24‐acetate (ALA‐24A) is an active triterpene that has antioxidant and anti‐inflammatory pharmacological properties. However, its effect and molecular mechanism on osteoarthritis progression have not been reported.

**Methods:**

IL‐1β‐induced chondrocyte injury model and monosodium iodoacetate (MIA)‐induced rat osteoarthritis model were used. The protective effects of ALA‐24A on osteoarthritis were evaluated by determining cell viability, extracellular matrix (ECM) degradation, inflammatory response and oxidative stress using CCK‐8 assay, Western blot, ELISA, and DCFH‐DA fluorescent probe. The severity and matrix degradation of articular cartilage were assessed by histopathological and immunohistochemical examination.

**Results:**

We found that ALA‐24A attenuated IL‐1β‐induced cell viability inhibition Moreover, ALA‐24A suppressed expression levels of ECM degradation‐related genes ADAMTS5 and MMP13, and promoted expression levels of ECM synthesis‐related genes Aggrecan and Collagen II. In addition, ALA‐24A treatment decreased reactive oxygen species (ROS) production and increased antioxidant enzymes (SOD, CAT, and GSH‐px) activities, while increased MDA levels. The inflammatory levels of NO, PGE2, TNF‐α, and IL‐6 were also reduced following treatment with ALA‐24A. Our data also revealed that ALA‐24A treatment triggered p‐AMPK upregulation and p‐mTOR downregulation. In rat osteoarthritis model, ALA‐24A treatment significantly alleviated the severity and matrix degradation of articular cartilage comparted with model group.

**Conclusions:**

Our findings suggested a protective role of ALA‐24A against osteoarthritis by inhibiting ROS and inflammatory response. Furthermore, ALA‐24A might be a promising therapeutic option for osteoarthritis treatment.

## Graphical abstract

The present study for the first time revealed that Alisol A 24‐acetate (ALA‐24A) attenuated cartilage degeneration and osteoarthritis progression by inhibiting reactive oxygen species and inflammatory response. Furthermore, this process might be related to regulate AMPK/mTOR pathway. Our findings might provide novel insights into ALA‐24A as a promising drug for osteoarthritis treatment.

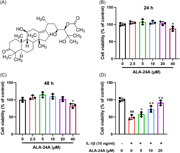

## 1 INTRODUCTION

Osteoarthritis is a common degenerative knee joint disease among elder people, and mainly characterized by articular cartilage degeneration, intra‐articular inflammation and subchondral bone remodeling.^1^, ^2^, ^3^ Evidence suggests that aging, genetics, female sex, obesity, and heavy work activities significantly increases osteoarthritis risk.^3^ With the increase of obesity and aging, the incidence of osteoarthritis has been sharply increased, and caused substantial physical disability and huge health care fee burden.^4^ The pathogenesis of osteoarthritis is complex. However, growing studies support unequivocal roles for excess inflammation and oxidative stress in the development of osteoarthritis. Accordingly, several anti‐inflammatory drugs, such as paracetamol and NSAIDs, have been considered as first‐line pharmacological treatment for osteoarthritis. But long‐term use of these drugs could cause serious adverse gastrointestinal reaction, renal and hepatic toxicity.^5^, ^6^ Therefore, developing new safe and therapeutic strategies is of great importance for osteoarthritis treatment.

**Figure 1:**
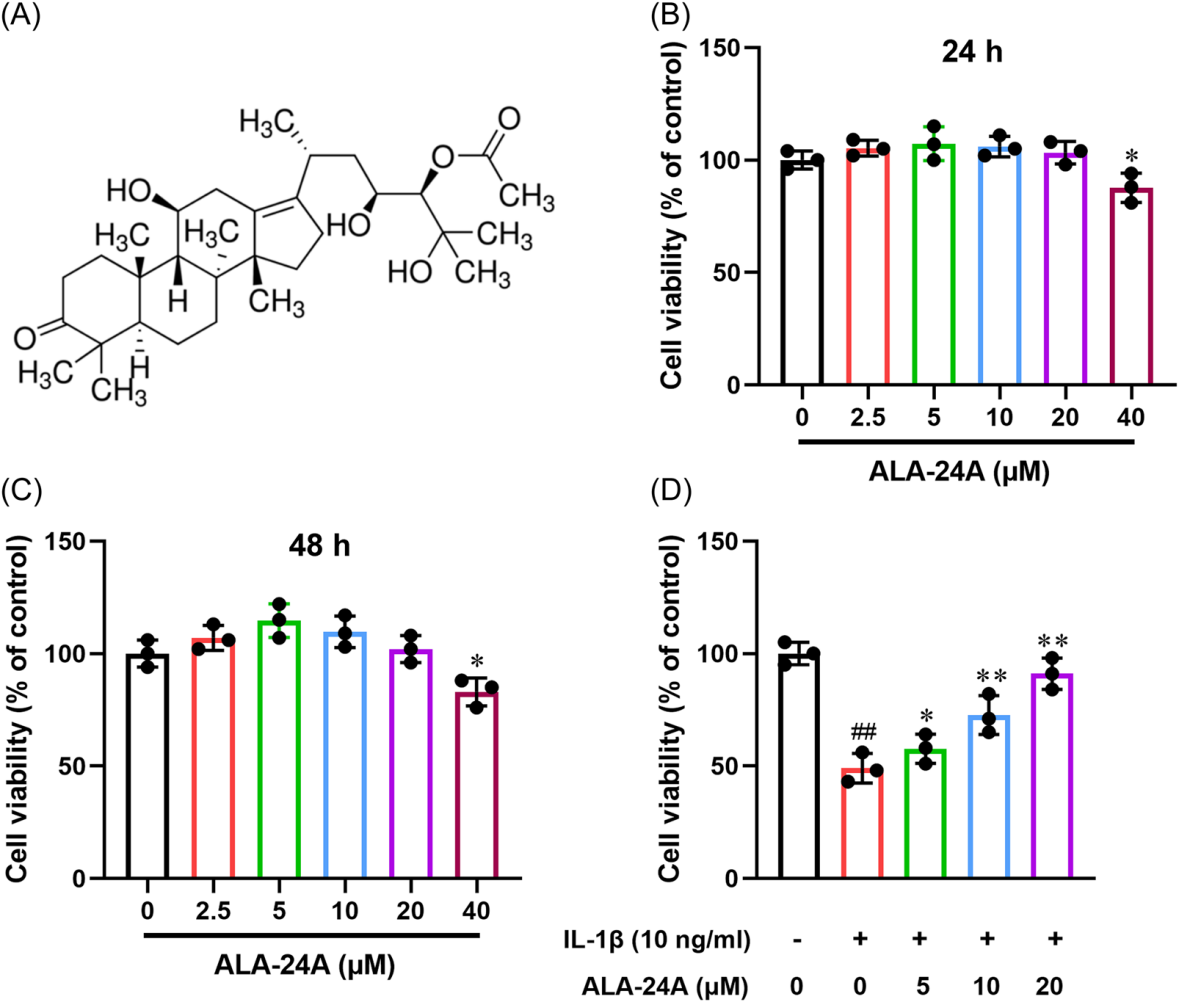
ALA‐24A attenuates the inhibitory effect of IL‐1β on the cell viability. (A) The chemical structure of ALA‐24A. (B–C) Chondrocytes were incubated with different concentrations (2.5, 5, 10, and 20 μM) of ALA‐24A for 24 h (B), and 48 h (C). The cell viability was determined by CCK‐8 assay at optical density 450 nm. (D) Chondrocytes were pretreated with 10 ng/mL recombinant IL‐1β for 1 h and then incubated with different concentrations of ALA‐24A for 24 h. The cell viability was determined by CCK‐8 assay at optical density 450 nm. Statistical significance was determined by the Student's *t* test, *n* = 3. ^##^ *p* < .01, compared with control group. *p < .05, **p < .01, compared with 10 ng/mL IL‐1β treatment group. ALA‐24A, Alisol A 24‐acetate.

Alisol A 24‐acetate (ALA‐24A, Figure 1A) is an active triterpene extracted from Alisma orientale (Sam.) Juz. (Alismataceae).^7^ Recent reports suggest that ALA‐24A possesses various pharmacological properties, including antioxidant, anti‐inflammatory, antitumor and neuroprotective activities. For instance, ALA‐24A was reported to suppress microglia and astrocytes proliferation, inflammatory response and neuron apoptosis through activating PI3K/AKT pathway.^8^ Using a nonalcoholic steatohepatitis mouse model, Wu et al.^9^ found that ALA‐24A treatment reduced the levels of oxidative stress and inflammation by regulating AMPK/mTOR pathway. In addition, ALA‐24A inhibited the apoptosis, tight junction degradation and inflammation induced by oxygen‐glucose deprivation in brain microvascular endothelial cells.^10^, ^11^ ALA‐24A has also been implicated in metabolic disorders via regulating accumulating lipolysis and glucose uptake, while reducing steatosis.^12^, ^13^, ^14^ However, the function of ALA‐24A in osteoarthritis remains unknown.

Considering the potent anti‐inflammatory and antioxidant properties of ALA‐24A, we hypothesized that ALA‐24A might exhibit a protective effect against osteoarthritis. To prove this hypothesis, we explored the effects ALA‐24A on extracellular matrix (ECM) degradation, inflammation as well as oxidative stress using IL‐1β‐stimulated chondrocyte model and monosodium iodoacetate (MIA)‐induced rat osteoarthritis model. Furthermore, we also explore the possible mechanisms related to this process.

## 2 MATERIALS AND METHODS

### 2.1 Isolation and culture of primary chondrocytes

The articular cartilage was excised from the knees and hips of 4‐week‐old Sprague Dawley rats, cut into 1 to 3 mm‐thick pieces and then digested with 0.25% trypsin for 30 min and 0.2% collagenase II for 5 h at 37°C. After centrifuging at 3000 rpm for 5 min, the chondrocytes in the supernatant were collected and cultured in the DMEM/F12 (HyClone). The third‐generation chondrocytes were used for subsequent studies.

### 2.2 Cell viability assay

Chondrocytes (6 × 10^3^ cells/well) were seeded into a 96‐well plate. After incubation overnight, chondrocytes were pretreated with 10 ng/mL recombinant rat IL‐1β (#I2393‐50UG, Sigma) for 1 h and then incubated with or without different concentrations of ALA‐24A (#PHL83836, purity ≥ 95.0%, Sigma) for indicated hours. Cell viability was determined by CCK‐8 assay (Beyotime). The optical density (450 nm) was detected by microplate reader (ELx800; BioTeck).

### 2.3 Analysis of NO, PGE2, inflammatory cytokines, and oxidative stress

Chondrocytes (6 × 10^3^ cells/well) were seeded into a 96‐well plate. After incubation overnight, chondrocytes were pretreated with IL‐1β (10 ng/mL) for 1 h and then incubated with different concentrations of ALA‐24A for another 24 h. Supernatant from the cell culture was collected. NO level was determined using Griess reagent (Beyotime). The levels of PEG2, TNF‐α, and IL‐6 in culture supernatant were detected using ELISA kits (R&D Systems). The supernatant levels of MDA, SOD, CAT, and GSH‐px were determined by ELISA kits (Beyotime).

### 2.4 Western blot

Treated chondrocytes were collected and lysed by RIPA (Beyotime). Equal protein samples (50 µg) separated by SDS‐PAGE and transferred onto PVDF membranes. Primary antibodies against Aggrecan, Collagen II, ADAMTS5, MMP13, p‐AMPK, AMPK, p‐mTOR, mTOR, and GAPDH were used in this study at a dilution of 1:1000. The results were visualized by a chemiluminescence detection system (Bio‐Rad).

### 2.5 Reactive oxygen species (ROS) detection

DCFH‐DA fluorescent probe (Beyotime) was used to detect intracellular ROS. Briefly, treated cells were incubated with 10 μM of DCFH‐DA at 37°C for 20 min and washed three times with serum‐free medium. The fluorescence intensity was evaluated by FACSCalibur flow cytometer (BD) or multimode microplate reader (Varioskan LUX 3020, Thermo Scientific) at 488 nm excitation and 525 nm emission wavelength.

### 2.6 Osteoarthritis rat model

Forty male Sprague Dawley rats (8‐week‐old, weighing 220–250 g, Vital River) were divided randomly into five groups (*n* = 8 rats/group): (1) Control group, rats were received intraarticular injection of normal saline; (2) Model group, MIA‐induced osteoarthritis rats were received intraperitoneal injection of normal saline every other day; (3) ALA‐24A‐low group, MIA‐induced osteoarthritis rats were received intraperitoneal injection of 5 mg/kg/day ALA‐24A every other day; (4) ALA‐24A‐middle group, MIA‐induced osteoarthritis rats were received intraperitoneal injection of 10 mg/kg/day ALA‐24A every other day; (5) ALA‐24A‐low group, MIA‐induced osteoarthritis rats were received intraperitoneal injection of 20 mg/kg/day ALA‐24A every other day. To induce the osteoarthritis model, rats were intraarticularly injected a single dose of MIA (Sigma, 2 mg MIA was dissolved in 50 μL of 0.9% saline) in the right posterior knee according to a previous study.^15^ After 4 weeks, rats were killed, and the knees were collected for subsequent pathological assessments. All protocols involved in animal experiment were approved by the Ethics Committee of The First Hospital of Putian City (NO. 20200361).

### 2.7 Histopathological and immunohistochemical examination

Tissue samples were fixed with 4% paraformaldehyde, paraffin‐embedded and sectioned (5 µm). After deparaffinization, sections were rehydrated and subjected to hematoxylin and eosin (HE) (Solarbio) or safranin O/fast green (Solarbio). The severity was independently scored by two researchers who did not know the group condition of the mice according to the Osteoarthritis Research Society International (OARSI) histopathology scoring system.^16^ Immunohistochemical staining was conducted on deparaffinized and hydrated knee joint tissue sections. After antigen retrieval, the sections were blocked, stained with anti‐MMP13 antibody (1:100) and secondary antibody. Finally, sections were stained with DAB hematoxylin.

### 2.8 Statistical analysis

Results were represented as mean ± SD. Statistical analysis was assessed by Student's *t* test and one‐way ANOVA using GraphPad Prism software version 6.0. *p* < .05 indicates a statistically significant difference.

## 3 RESULTS

### 3.1 ALA‐24A attenuates the inhibitory effect of IL‐1β on the cell viability

CCK‐8 assay results revealed that ALA‐24A had no significant inhibitory influence on chondrocytes at low concentration (2.5, 5, 10, and 20 μM) at 24 and 48 h (Figure 1B,C). High dose of ALA‐24A (40 μM) showed a slight inhibition on chondrocyte viability. Cell viability in IL‐1β treatment group was apparent lower than control group (Figure 1D). Meanwhile, ALA‐24A concentration‐dependently increased the viability in IL‐1β‐pretreated chondrocytes. Overall, ALA‐24A alleviates IL‐1β‐induced cell injury in chondrocytes.

### 3.2 ALA‐24A suppresses IL‐1β‐induced ECM degradation in chondrocytes

**Figure 2:**
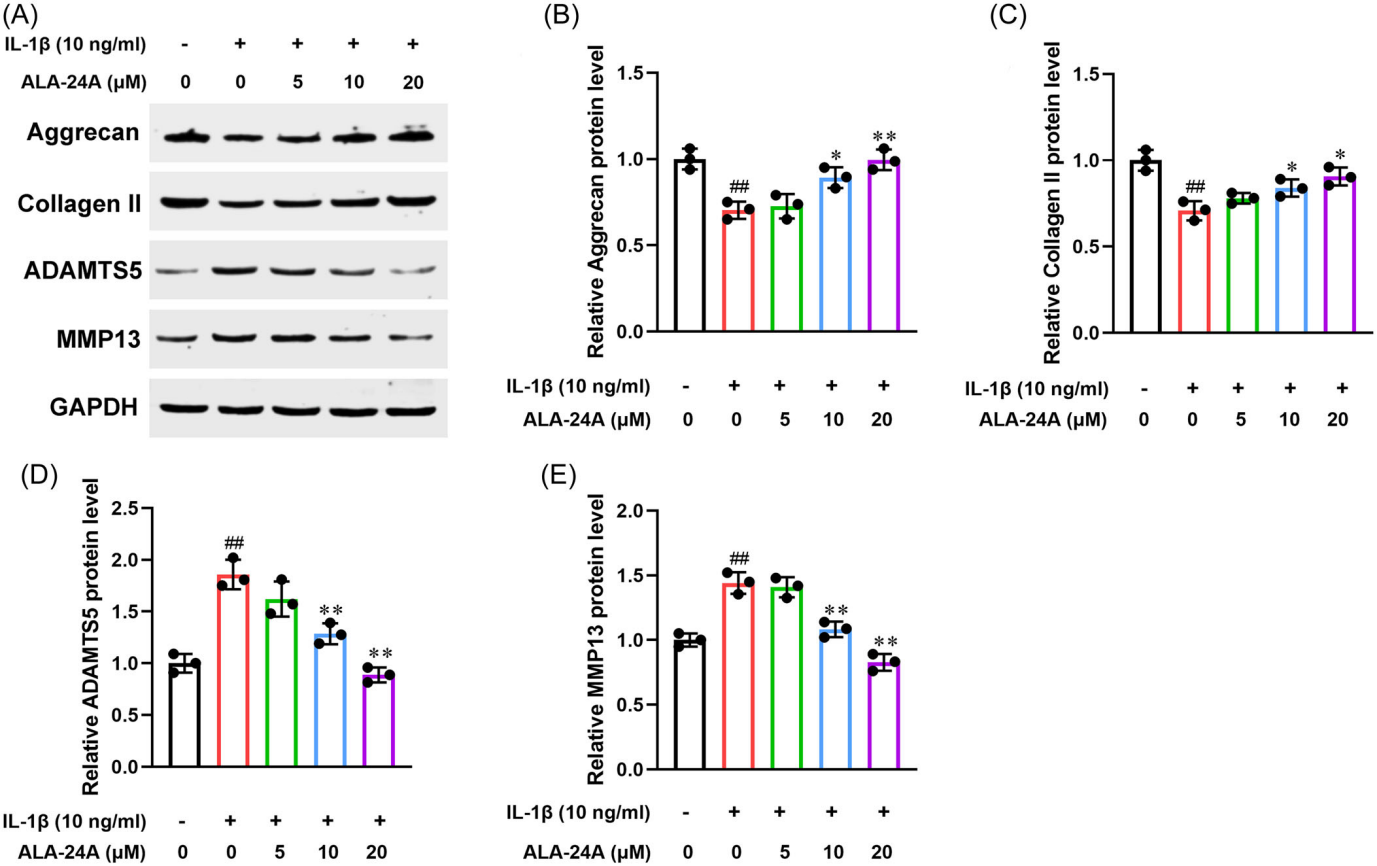
ALA‐24A suppresses IL‐1β‐induced ECM degradation in chondrocytes. Western blot was performed to evaluate the effects of ALA‐24A on the levels of ECM degradation and synthesis‐related proteins in IL‐1β‐induced osteoarthritis chondrocytes. (A) Expression levels of Aggrecan, Collagen II, ADAMTS5, and MMP13 were determined by Western blot. (B) Quantitative analysis of Aggrecan. (C) Quantitative analysis of Collagen II. (D) Quantitative analysis of ADAMTS5. (E) Quantitative analysis of MMP13. Statistical significance was determined by the Student's *t* test, *n* = 3. ^##^ *p* < .01, compared with control group. *p < .05, **p < .01, compared with 10 ng/mL IL‐1β treatment group. ALA‐24A, Alisol A 24‐acetate.

Next, we explored whether ALA‐24A could affect ECM metabolism using Western blot. IL‐1β treatment significantly reduced the levels of ECM synthesis‐related genes Aggrecan and Collagen II, and obviously increased the levels of ECM degradation‐related genes ADAMTS5 and MMP13. Compared with IL‐1β group, ALA‐24A treatment partially reversed IL‐1β‐induced upregulation of Aggrecan and Collagen II expression, and downregulation of ADAMTS5 and MMP13 expression in chondrocytes (Figure 2A–E).

### 3.3 ALA‐24A inhibits ROS and increased the activities of antioxidant enzymes

**Figure 3:**
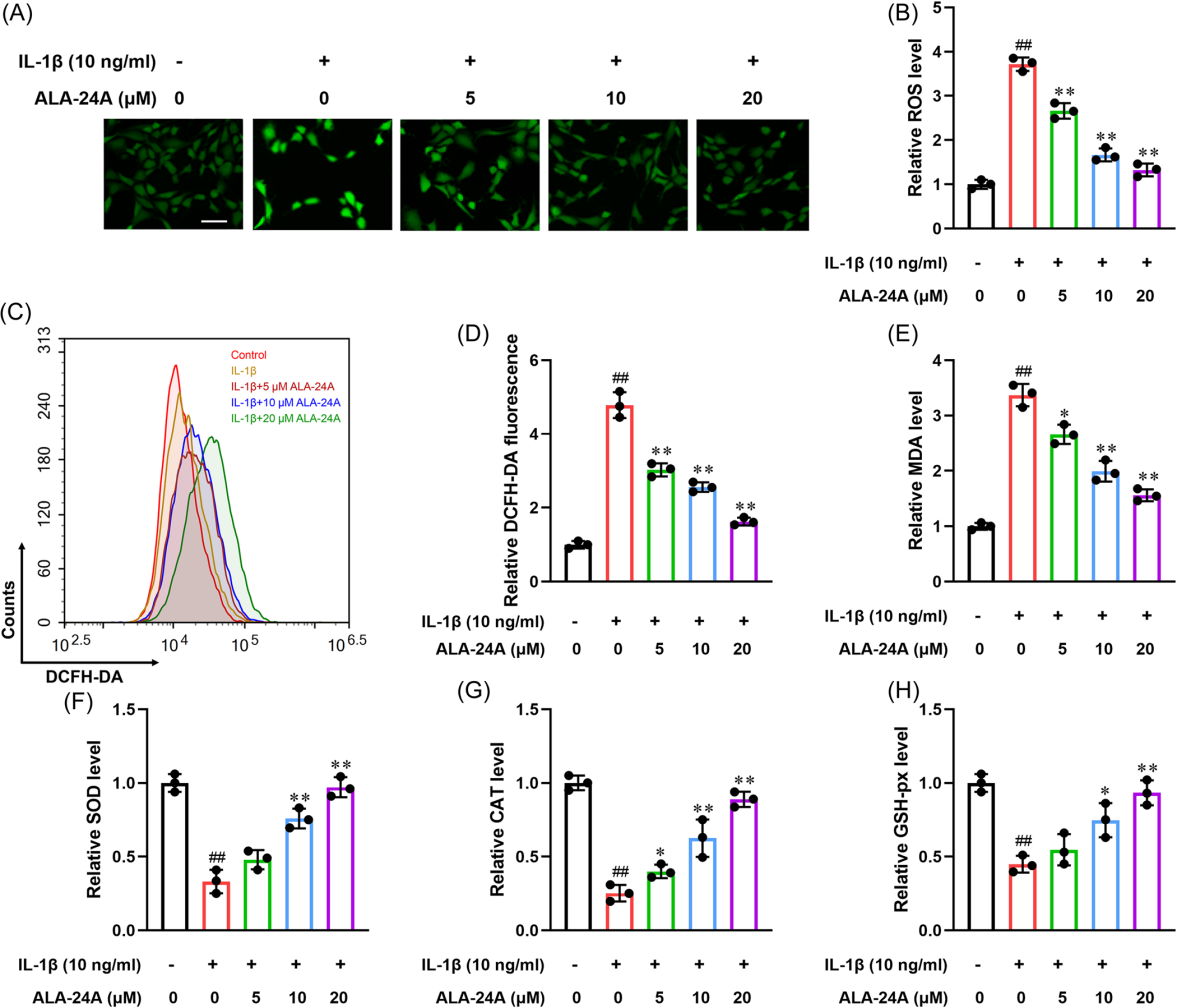
ALA‐24A inhibits ROS and increased the activities of antioxidant enzymes. (A) Intracellular ROS were detected by ROS Assay Kit using DCFH‐DA fluorescent probe. Scale bar, 100 μm. (B) Quantitative analysis of the fluorescence intensity. (C) ROS levels were measured using a DCFH‐DA probe and flow cytometry analysis. (D) Quantitative analysis of DCFH‐DA fluorescence. (E‐H) Chondrocytes were pretreated with IL‐1β (10 ng/mL) for 1 h and then incubated with different concentrations of ALA‐24A for another 24 h. Supernatant from the cell culture was collected. The supernatant levels of MDA (E), SOD (F), CAT (G) and GSH‐px (H) were detected using ELISA kits according to the manufacturer's instructions. Statistical significance was determined by the Student's *t* test, *n* = 3. ^##^ *p* < .01, compared with control group. *p < .05, **p < .01, compared with 10 ng/mL IL‐1β treatment group. ALA‐24A, Alisol A 24‐acetate; ROS, reactive oxygen species.

Compared with control group, DCFH‐DA fluorescent probe analysis demonstrated that IL‐1β significantly upregulated cellular ROS production, while ALA‐24A decreased IL‐1β‐induced ROS production in a dose‐dependent manner (Figure 3A–D). Additionally, we noted an obvious increase in MDA levels with IL‐1β stimulation but a remarkable decrease after co‐incubation with ALA‐24A (Figure 3E). In contrast, IL‐1β reduced the activities of antioxidant enzymes (SOD, CAT, and GSH‐px), but this effect was reversed by ALA‐24A (Figure 3F–H).

### 3.4 ALA‐24A inhibits inflammatory response

**Figure 4:**
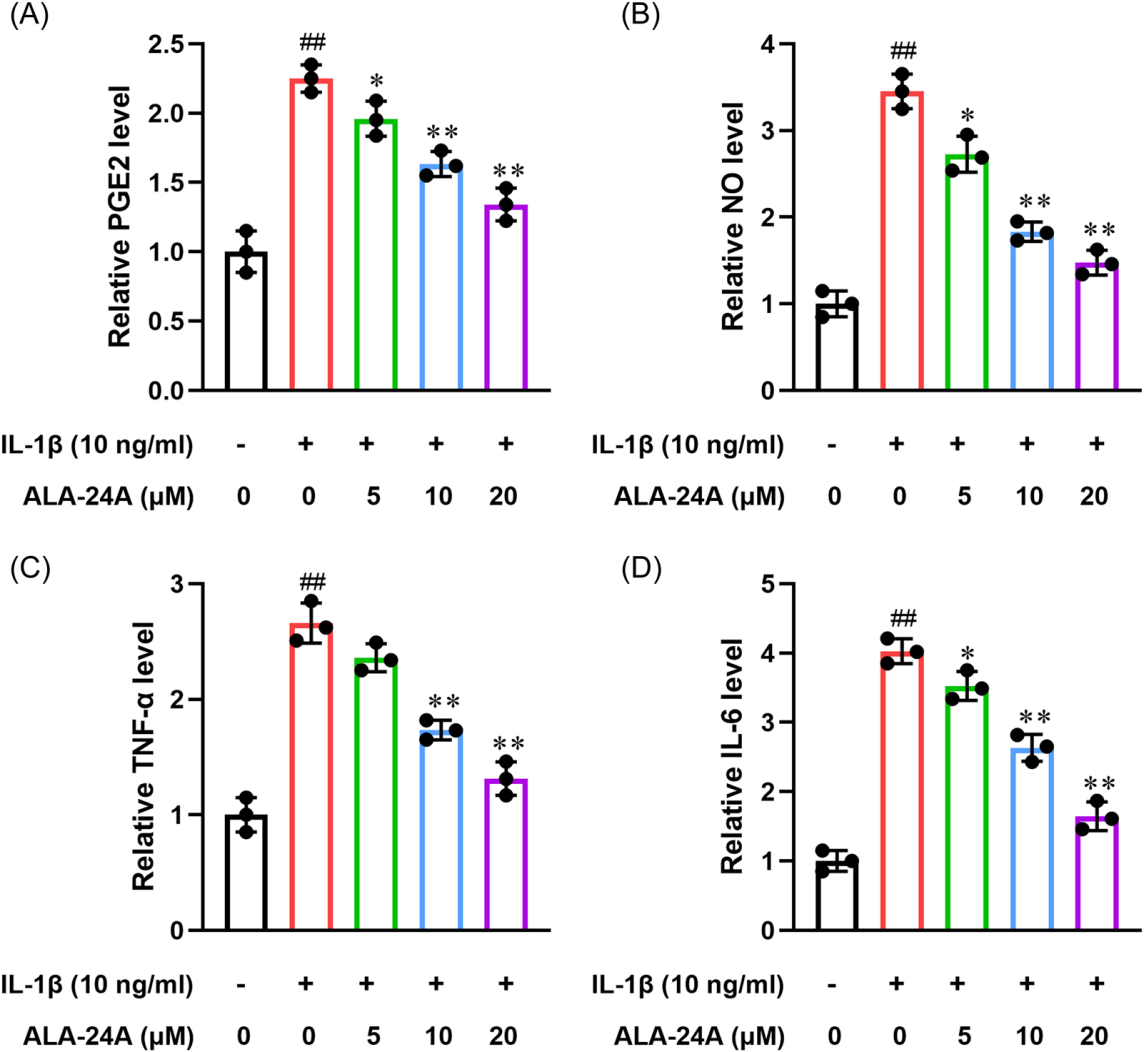
ALA‐24A inhibits inflammatory response. Chondrocytes were pretreated with IL‐1β (10 ng/mL) for 1 h and then incubated with different concentrations of ALA‐24A for another 24 h. Supernatant from the cell culture was collected. (A) NO production was analyzed using Griess reagent. (B‐D) The levels of PEG2 (B), TNF‐α (C), and IL‐6 (D) in culture supernatant were detected using ELISA kits according to the manufacturer's instructions. Statistical significance was determined by the Student's *t* test, *n* = 3. ^##^ *p* < .01, compared with control group. **p < .01, compared with 10 ng/mL IL‐1β treatment group. ALA‐24A, Alisol A 24‐acetate.

Subsequently, we investigated the influence of ALA‐24A on the inflammatory response. Griess reaction and ELISA assay results showed that the levels of NO, PGE2, TNF‐α, and IL‐6 were obviously higher in IL‐1β group than control group (Figure 4A–D). However, IL‐1β‐induced inflammatory response were diminished after treatment with ALA‐24A.

### 3.5 ALA‐24A inhibits the activation AMPK/mTOR pathway

**Figure 5:**
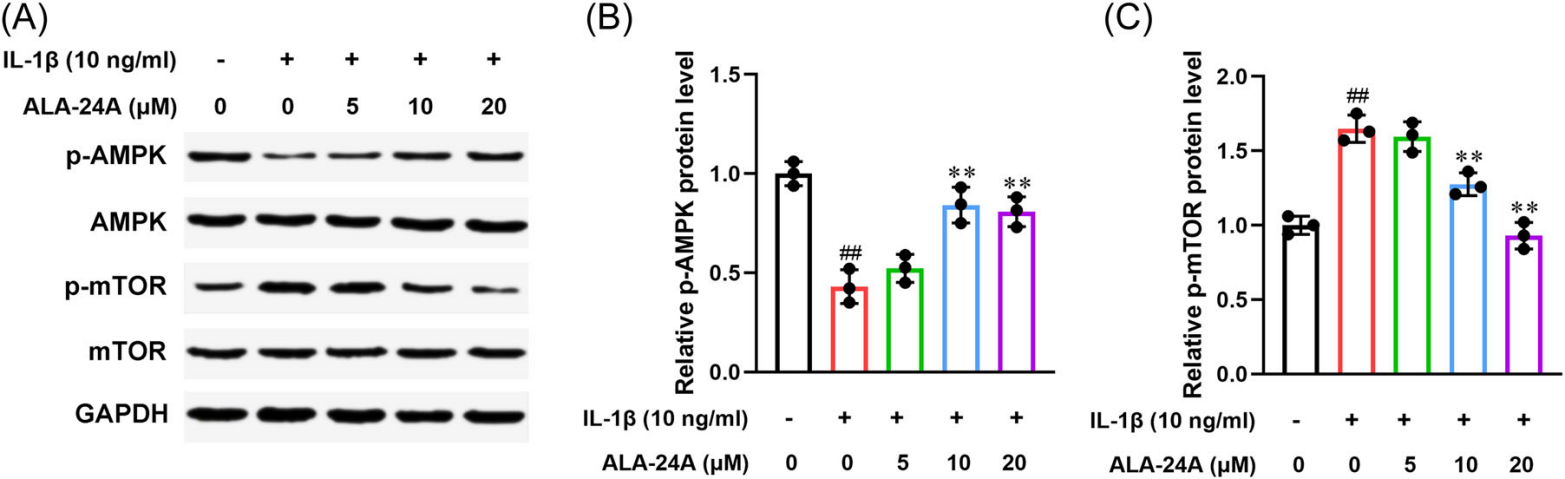
ALA‐24A inhibits the activation AMPK/mTOR pathway. Western blot was performed to evaluate the effects of ALA‐24A on AMPK/mTOR pathway in IL‐1β‐induced osteoarthritis chondrocytes. (A) Expression levels of p‐AMPK, AMPK, p‐mTOR, and mTOR were determined by Western blot. (B) Quantitative analysis of p‐AMPK. (B) Quantitative analysis of p‐mTOR. Statistical significance was determined by the Student's *t* test, *n* = 3. ^##^ *p* < .01, compared with control group. **p < .01, compared with 10 ng/mL IL‐1β treatment group. ALA‐24A, Alisol A 24‐acetate.

Previous studies showed that AMPK/mTOR pathway had a major role in osteoarthritis progression.^17^, ^18^, ^19^ We assumed that ALA‐24A exhibited its protective role against osteoarthritis by regulating AMPK/mTOR pathway. We performed Western blot to prove this hypothesis. As showed in Figure 5A–C, IL‐1β treatment caused p‐AMPK upregulation and p‐mTOR downregulation compared with control group, but did not influence AMPK and mTOR expression. There was no obvious impact of IL‐1β on AMPK and mTOR. However, ALA‐24A treatment reversed the regulatory effects of IL‐1β.

### 3.6 ALA‐24A alleviates osteoarthritis progression in vivo

**Figure 6:**
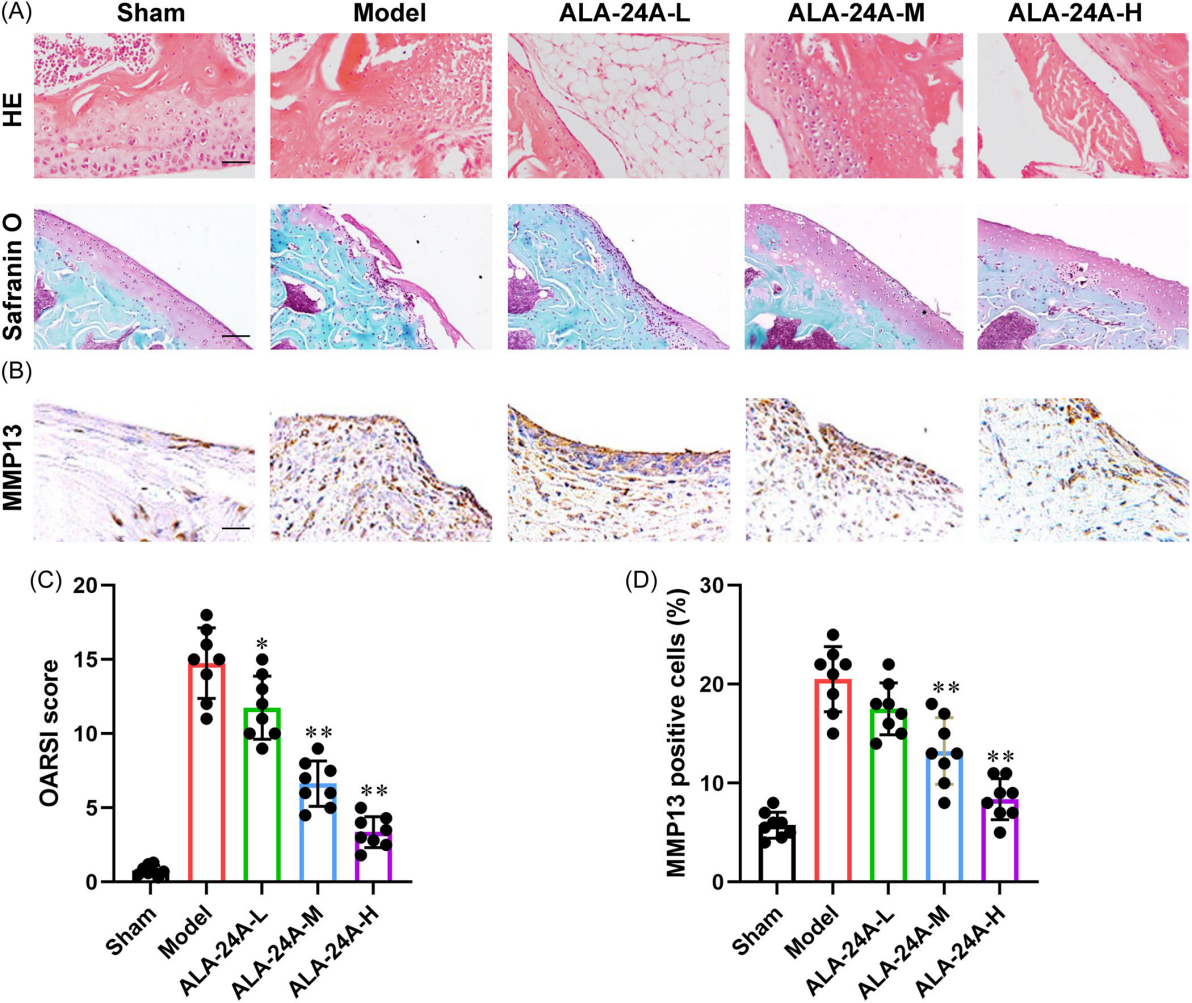
ALA‐24A alleviates osteoarthritis progression in vivo. Forty eight‐week‐old male Sprague‐Dawley rats were randomly divided into five groups (*n* = 8 rats per group). Different doses of ALA‐24A were intraperitoneally administrated in MIA‐induced osteoarthritis rats every other day. All rats were euthanized after 4 weeks of treatment. (A) Histopathological examination of rat knee joints by HE and safranine O staining (×200). (B) MMP13 expression of rat knee joints were detected by IHC staining (×200). (C) The OARSI scores. (D) Quantitative analysis of MMP13 by the IHC staining. Statistical significance was determined by the Student's *t* test, n = 8. ^##^ *p* < .01, compared with sham group. *p < .05, **p < .01, compared with model group. ALA‐24A, Alisol A 24‐acetate.

To further study whether ALA‐24A has protective function on osteoarthritis, osteoarthritis rat model was constructed and then treated with different doses of ALA‐24A for 4 weeks. Compared with sham group, the knee joints of model group exhibited surface irregularity, surface cleft and loss of articular cartilage, but ALA‐24A treatment alleviated the severity and matrix degradation (Figure 6A). Besides, we observed a reduced OARSI scores in ALA‐24A treatment group, indicating that ALA‐24A reduced the cartilage damage in osteoarthritis (Figure 6C). As shown in Figure 6B,D, IHC staining confirmed that ALA‐24A treatment reduced MMP3 expression in cartilage tissue. Our findings imply that ALA‐24A treatment ameliorates osteoarthritis progression.

## 4 DISCUSSION

Osteoarthritis is a common chronic inflammation disease. All current interventions including surgical treatment aim to confer temporary pain and swelling relief, but cannot completely reverse the disease progression.^20^, ^21^, ^22^ Natural products obtained from diet and plants have become a major resource of new drug discovery, considering their potential effectiveness and low toxicity profiles. In recent studies, researchers demonstrated that many anti‐inflammatory natural compounds showed an excellent protective effect in osteoarthritis. For example, arctigenin is an active lignan of *Arctium lappa* that was reported to suppress inflammatory response in diabetic kidney disease, acute colitis and psoriasis.^23^, ^24^, ^25^ In osteoarthritis, Tang et al.^26^ found that arctigenin treatment could decrease the inflammatory cytokines and ECM degradation through PI3K/Akt/NF‐κB pathway. Isorhamnetin is a monomethoxyflavone that showed a distinct anti‐inflammatory activity in nonalcoholic steatohepatitis, reflux esophagitis and acute liver injury.^27^, ^28^, ^29^ Several studies discovered that isorhamnetin could ameliorate inflammatory response and articular cartilage damage, and attenuate osteoarthritis progression.^30^, ^31^, ^32^ ALA‐24A has also been found to inhibit inflammation in many diseases, yet its role in osteoarthritis remains unclear.^8^, ^9^, ^10^, ^11^ In the present study, we found that ALA‐24A treatment could inhibited IL‐1β‐induced ECM degradation and inflammation. Importantly, only slight inhibition on chondrocyte viability was observed when treated with high dose of ALA‐24A (40 μM), indicating that ALA‐24A might be a relative safe and low‐toxic bioactive component for osteoarthritis treatment.

Another potential contributing factor in the progression of osteoarthritis is the oxidative stress. Increased ROS level and decreased antioxidant activities have been observed in human osteoarthritis cartilage.^33^, ^34^, ^35^ When cellular antioxidant defense system cannot effectively eliminate massively accumulated ROS, the body will produce excessive oxidation intermediates, such as NO, which can induce synovial cell death by regulating mitochondrial function and increasing the expression of p‐53.^36^, ^37^ This imbalance will eventually lead to the loss of homeostasis in articular cartilage, directly or indirectly promotes the production of inflammatory reactions and chondrocyte death.^38^ In IL‐1β‐induced osteoarthritis, we found that the level of ROS was increased while the activities of antioxidant enzymes were decreased. However, ALA‐24A treatment reversed this tendency. These findings suggest that ALA‐24A can alleviate osteoarthritis progression by suppressing oxidative stress.

AMPK and mTOR are two important serine/threonine kinases that involved in sensing the availability of nutrients and energy and regulation of cell growth.^39^ They have been broadly implicated in the regulation of cell proliferation, apoptosis and autophagy in physiological and pathological conditions.^40^, ^41^, ^42^ Although the two pathways are interlinked, but AMPK activation always blocks the phosphorylation of mTOR signaling pathway.^43^, ^44^ In osteoarthritis, many natural products were found to attenuate cartilage degeneration and osteoarthritis progression via regulating AMPK/mTOR pathway, such as metformin, active vitamin D and sinensetin.^17^, ^18^, ^45^, ^46^ Consistent with these findings, we observed that ALA‐24A treatment triggered p‐AMPK upregulation and p‐mTOR downregulation in vitro, indicating that AMPK/mTOR pathway might be responsible for the protective effect of ALA‐24A in osteoarthritis.

In summary, our study revealed that ALA‐24A attenuated cartilage degeneration and osteoarthritis progression by inhibiting oxidative stress and inflammatory response. Furthermore, this process might be related to regulate AMPK/mTOR pathway. Our findings suggest ALA‐24A as a potential therapeutic option for osteoarthritis treatment. However, the current research has certain limitations. For instance, we failed to explain the detailed mechanism through which ALA‐24A regulate AMPK/mTOR pathway and what's the direct target of ALA‐24A in osteoarthritis. Further studies are needed to solve this issue using chemical biology and molecular biology technologies, including molecular probes, mass spectrometry, pull‐down experiments and molecular docking.

## AUTHOR CONTRIBUTIONS

Guosong Xu and Yinping Liu conceived and designed the study. Guosong Xu, Zhensen He, and Yinping Liu performed the experiments and collected the data. Guosong Xu and Zhensen He analyzed the data. Guosong Xu and Zhensen He wrote the manuscript. Yinping Liu revised the manuscript.

## CONFLICT OF INTEREST STATEMENT

The authors declare no conflict of interest.

## Data Availability

The data that support the findings of this study are available from the corresponding author upon reasonable request.
